# Beta-2-glycoprotein I as a biomarker for sepsis in critically ill patients in the intensive care unit: a prospective cohort study

**DOI:** 10.1186/s13054-020-03066-3

**Published:** 2020-06-15

**Authors:** Irene T. Schrijver, Hans Kemperman, Mark Roest, Jozef Kesecioglu, Dylan W. de Lange

**Affiliations:** 1 Department of Intensive Care Medicine, University Medical Centre, University of Utrecht, Heidelberglaan 100, 3584 CX Utrecht, The Netherlands; 2 Department of Clinical Chemistry and Hematology, Central Laboratory (CDL), University Medical Centre, University of Utrecht, Heidelberglaan 100, 3584 CX Utrecht, The Netherlands; 3 Synapse B.V., Maastricht, The Netherlands; 4 Dutch Poisons Information Center (DPIC), University Medical Center, University of Utrecht, Heidelberglaan 100, 3584 CX Utrecht, The Netherlands

**Keywords:** B2GPI, Biomarkers, Sepsis, Critical care, ICU, SIRS, Apolipoprotein H

To the editor,

It is difficult to diagnose sepsis in critically ill patients admitted to the intensive care unit (ICU). A biomarker could help in sepsis identification and guide antibiotic use. A prognostic biomarker that identifies high-risk patients for the development of sepsis or nosocomial infections could help prevent those outcomes. However, current sepsis biomarkers often portray systemic inflammation and are unspecific to infection [1].

The complement system is essential for defending against infections, yet it can also contribute to severe sepsis outcomes [2]. A potential complement regulator is beta-2-glycoprotein I (B2GPI). B2GPI, also known as apolipoprotein H, exerts a complement control after binding to specific surfaces such as apoptotic cells and bacteria. After the conformational change of B2GPI, monocytes can clear B2GPI, resulting in lower B2GPI plasma levels. Supporting this, previous studies found lower levels of B2GPI after lipopolysaccharide (the main component of the outer membrane of bacteria) infusion in humans and male mice [3, 4]. Therefore, we hypothesized that B2GPI levels can differentiate between sepsis and non-infectious critically ill patients. To test this and to determine the discriminative and prospective value of B2GPI as a sepsis biomarker, we conducted a prospective study.

We included 313 critically ill adult patients (defined as two or more systemic inflammatory response syndrome (SIRS) criteria upon admission) with an anticipated ICU stay of more than 24 h (Table 1) [5]. In 48 h following ICU admission, blood was sampled twice to measure the maximum level of B2GPI using a semi-automated sandwich ELISA on a Freedom EVO platform (Tecan) with goat anti-human beta-2-glycoprotein-1 and HRP-coupled goat-human beta-2-glycoprotein-1 antibodies (both Cedarlane). Supersignal West Pico Chemiluminescent substrate (Thermo Scientific) was added, and the luminescence signal was measured using a Spectramax-L microplate reader (MDS Analytical). The outcomes “no sepsis,” “sepsis,” and “septic shock” were defined according to the sepsis-3 criteria. The outcome “proven infection” was derived from Centers for Disease Control (CDC) algorithms. Statistical analyses were performed using SPSS version 21.0 and R version 3.6.1.

**Table 1:** Descriptive characteristics, medians (IQR) or *N* (%)

Characteristic	No sepsis	Sepsis	Septic shock	*P* value
Number of patients	180	75	47	n/a
Gender, male	113 (62%)	47 (38%)	33 (67%)	0.778
Age (years)	60 (23)	61 (28)	65 (25)	0.656
Diagnosis at admission				
Trauma	8 (4%)	10 (13%)	5 (10%)	0.028*
Infection (suspicion of)	40 (22%)	20 (26%)	19 (40%)	
Post surgery	35 (19%)	11 (15%)	6 (12%)	
Others	97 (54%)	35 (46%)	18 (38%)	
Severity of illness (at admission)				
Mechanical ventilation	104 (86%)	43 (78%)	26 (65%)	< 0.001*
APACHE IV score	70 (44)	74 (38)	93 (44)	0.003*
SOFA score	6 (5)	5 (5)	11 (5)	< 0.001*
30-day mortality	39 (21%)	19 (25%)	17 (34%)	0.157
ICU stay (days)	10 (10)	8 (10)	8 (9)	0.267
Gram-negative infection	25 (14%)	25 (33%)	22 (45%)	< 0.001*
β2 Glycoprotein-1 (μg/mL)	198 (313)	165 (195)	129 (149)	< 0.001*
CRP (mg/L)	187 (166)	245 (200)	290 (168)	< 0.001*
Procalcitonin	1.0 (3.0)	2.9 (8.1)	4.5 (35.8)	< 0.001*
Leukocytes (× 109/L)	16 (9)	16 (10)	16 (10)	0.937

Mechanical ventilation direct upon admission; *IQR* interquartile range, *MPO* myeloperoxidase, *APACHE IV* Acute Physiology and Chronic Health Evaluation IV severity of illness model, *CRP* C-reactive protein, *SOFA* Sequential Organ Failure Assessment. Baseline characteristic comparisons were made using Mann-Whitney *U*, chi-square, or Kruskal-Wallis tests for skewed variables and Student’s *T* test or chi-square test for normal distributed variables. *P < 0.05

We found that B2GPI levels were significantly lower in patients with sepsis compared with patients without sepsis. Patients with a septic shock had lower B2GPI levels compared with patients with solely sepsis (Fig. 1a). Patients with an infection had lower levels of B2GPI (Fig. 1b). Patients within the highest tertile of B2GPI levels developed 44% fewer infections compared with the lowest tertile (*P* = 0.007, Fig. 1d) in the 10-day follow-up. There was no significant difference in B2GPI levels between survivors and non-survivors (Fig. 1c) and between gram-positive and gram-negative sepsis (162 [IQR 87–275] vs 131 [IQR 96–221]).

**Fig. 1:**
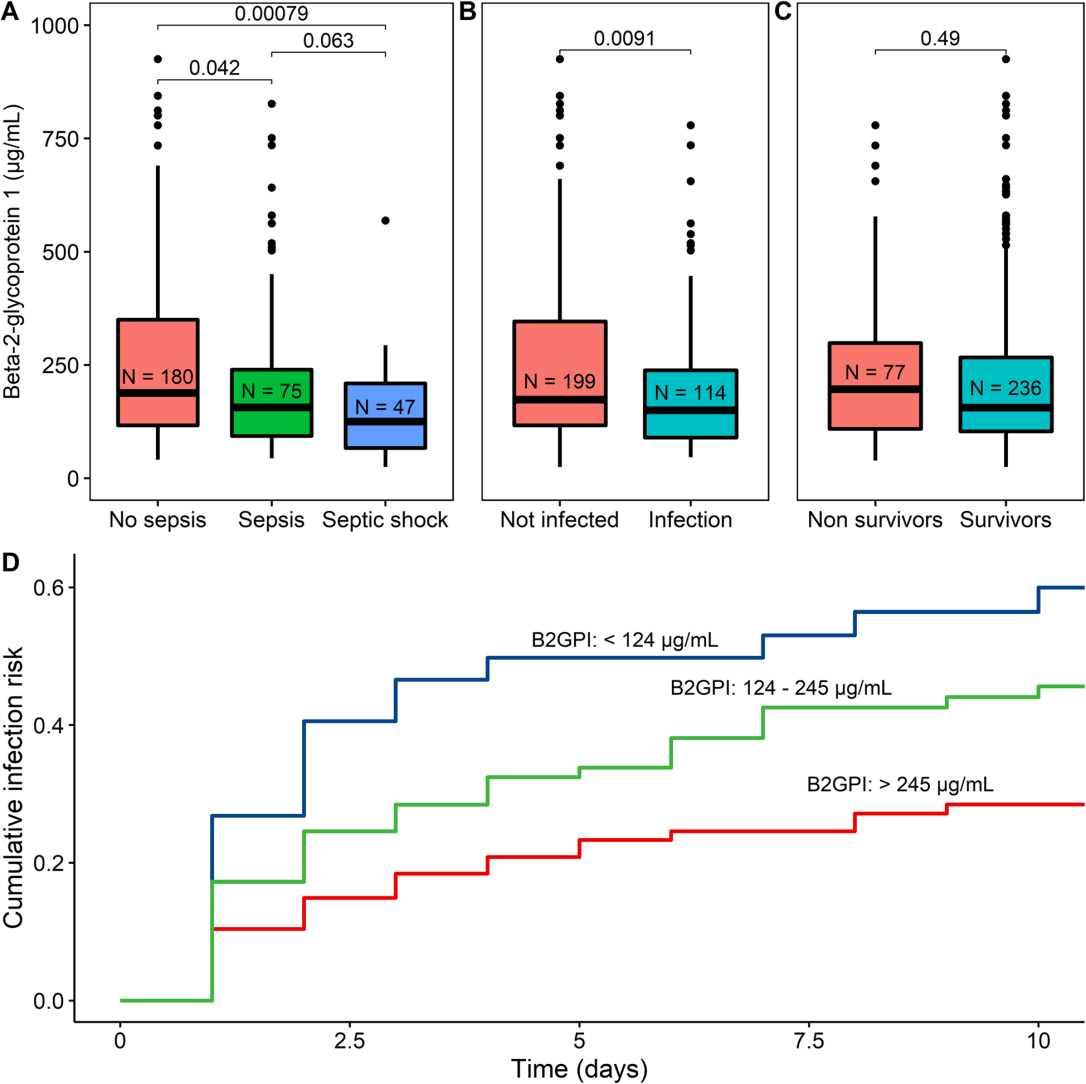
Beta-2-glycoprotein-1 (B2GPI) levels in different states of inflammation. **a** The correlation of the B2GPI level with no sepsis, sepsis, and septic shock was assessed using the Kruskal-Wallis and the Mann-Whitney *U* tests. **b** The B2GPI level in non-infected and infected patients assessed using the Mann-Whitney *U* test. **c** The B2GPI level in non-survivors and survivors assessed using the Mann-Whitney *U* test. **d** Development of infection during ICU stay depended on beta-2-glycoprotein-1 (B2GPI, μg/mL). The cohort was divided into three equal tertiles based on the B2GPI level. The relation between the development of infection and the different tertiles was calculated by applying the Kaplan-Meier method. Statistical differences between survival curves were analyzed using Cox proportional hazards regression analysis. Highest levels of B2GPI differ significantly from the lowest levels of B2GPI (*P* = 0.007)

We showed that B2GPI could differentiate between patients with and without sepsis. Moreover, patients with lower B2GPI levels in the first 48 h developed more nosocomial infections. This suggests that B2GPI may be a novel biomarker for both diagnosing sepsis and predicting nosocomial infections. This study took place in a general ICU; therefore, it seems feasible that our results could be generalizable to other ICUs.

Most biomarkers spike in sepsis; therefore, the lower B2GPI levels we found are relatively unique. Other biomarkers that decrease in sepsis are the inter-alpha inhibitor protein, lysophosphatidylcholine, and uric acid [1, 6]. However, currently, they are not used in clinical practice.

In summary, this is the first study that showed the value of the B2GPI biomarker in ICU patients with both a discriminative (for sepsis) and predictive (for nosocomial infections) role. Potentially, B2GPI can be helpful in diagnosing sepsis and stratifying ICU patients for infection risk.

## Data Availability

The datasets used and/or analyzed during the current study are available from the corresponding author on reasonable request.

## Abbreviations

B2GPI: Beta-2-glycoprotein I
CDC: Centers for Disease Control
ICU: Intensive care unit
SIRS: Systemic inflammatory response syndrome
